# Activation of circulating γδ T cells in pediatric IgA vasculitis nephritis links the IL-17 A+ subset to renal risk

**DOI:** 10.1038/s41598-026-57972-7

**Published:** 2026-06-15

**Authors:** Changqiang Yang, Yue Song

**Affiliations:** 1 Department of Cardiology, The First Affiliated Hospital of Chengdu Medical College, Chengdu, 610500 Sichuan China; 2 Department of Pediatrics, The First Affiliated Hospital of Chengdu Medical College, Chengdu, 610500 Sichuan China; 3 Department of Clinical medicine, The Chengdu Medical College, Chengdu, Sichuan China

**Keywords:** IgA vasculitis, Nephritis, γδ T cells, Interleukin-17A, Proteinuria, Diseases, Immunology, Nephrology

## Abstract

Renal involvement is a key determinant of prognosis in IgA vasculitis (IgAV), yet its underlying immunologic mechanisms remain incompletely elucidated. The role of γδ T cells and their subsets requires clarification. The study enrolled 88 children between September 1, 2023, and September 1, 2025, categorized into three groups: IgAV with nephritis (IgAVN, *n* = 39), IgAV without nephritis (IgAVwoN, *n* = 36), and healthy controls (HC, *n* = 13). Flow cytometry was used to assess the percentage of peripheral blood γδ T cells, their surface phenotype (CD69, NKG2D, TLR4), and intracellular cytokines (IL-17 A, IL-10, TNF-α). Multivariable logistic regression identified independent risk factors, receiver operating characteristic curve analysis evaluated diagnostic performance, and correlation with 24-hour proteinuria was assessed. Patients with IgAVN exhibited a significantly increased frequency of total γδ T cells and an expanded CD69⁺ activated subset. TLR4 expression on γδ T cells was upregulated in IgAV patients compared to HC group, but did not differ significantly between the IgAVN and IgAVwoN groups. The IL-17 A producing γδ T cell subset was identified as an independent risk factor for IgAVN (OR = 1.202, 95% CI 1.014–1.424, *P* = 0.034). Its frequency positively correlated with 24-hour proteinuria levels (*r* = 0.575, *P* < 0.01). This parameter demonstrated high specificity (97.22%) and moderate diagnostic accuracy (AUC = 0.673) for distinguishing IgAVN. IL-17 A⁺ γδ T cells were an independent risk factor for IgAVN and correlated with renal injury severity. While Th17 cells are the main IL-17 source, γδ T cells may represent an additional source. Further investigation is needed.

**Supplementary Information:**

The online version contains supplementary material available at https://doi.org/10.1038/s41598-026-57972-7.

## Introduction

IgA vasculitis (IgAV, previously known as Henoch–Schönlein purpura) is the most prevalent vasculitis of childhood, with an annual incidence of 3–26 cases per million children^1^. Patients typically present with palpable purpura, arthritis, and gastrointestinal symptoms. Renal impairment occurs in 18%-81% of patients^2,3^. Although most children with IgAV nephritis (IgAVN) have a favorable prognosis, the disease remains a notable cause of long-term morbidity and mortality, with 1–2% progressing to renal failure^4^.

The pathogenesis of IgAV remains incompletely understood. Multifactorial mechanisms are implicated, with immune dysfunction regarded as a key component^5–10^. Alterations in T cell function, along with cytokine and inflammatory mediator activity, contribute to immunological disturbances. In particular, the pro‑inflammatory cytokine Interleukin-17 A (IL‑17 A) has been implicated in the immunopathology of various glomerular diseases, where it promotes neutrophil recruitment and podocyte injury^5–9^. Accumulating evidence has highlighted the role of specific T cell subsets in IgAV pathogenesis. Elevated serum levels of IL-4 and IL-17 have been observed in pediatric IgAV patients, with TLR6 expression on monocytes positively correlated with both Th2- and Th17-related cytokines, suggesting the involvement of these T cell subsets^11^. A recent integrated bioinformatic analysis further confirmed the over-differentiation of Th2, Th17, and T follicular helper (Tfh) cells in IgAVN^12^. Additionally, a systematic review has summarized the abnormalities of Th cell subsets in this disorder, including Th2 and Th17 cells^13^. However, beyond the well-recognized roles of these helper T cells, the involvement of other T cell subsets in IgAV/IgAVN pathogenesis remains to be fully elucidated.

γδ T cells (1–10% of blood CD3^+^ T cells)^14^ bridge innate and adaptive immunity, maintaining homeostasis and contributing to immunopathology in infection, cancer, and autoimmunity^15^. They include subsets capable of rapidly producing cytokines such as IL-17A. Growing evidence links γδ T cells to kidney immunopathology, including elevated levels in IgA nephropathy^16^. Guo et al.^17^proposed that γδ T cell driven imbalance may participate in IgAV. Unlike the well-characterized roles of Th2 and Th17 cells, the involvement of γδ T cells, especially the IL-17A-producing subset, in the pathogenesis of renal injury in IgAVN has yet to be determined. Here, we conducted an initial investigation into γδ T cell associated immune dysregulation in patients with IgAV and IgAVN, with a focus on the IL‑17A producing subset and its potential link to clinical nephritis.

## Methods

### Study population

This study was conducted at a tertiary hospital in Chengdu, China. Patients and healthy controls were recruited between September 1, 2023, and September 1, 2025. The study protocol adhered to the principles of the Declaration of Helsinki and was approved by the Ethics committee of the first affiliated hospital of Chengdu medical college (Approval No. 2023CYFYIRB-BA-007). Written informed consent was obtained from the legal guardians of all participants.

Eligible patients were children under 14 years of age newly diagnosed with IgAV based on the EULAR/PRINTO/PRES 2008 classification^17^. Exclusion criteria were: (1) concurrent inflammatory or renal diseases; (2) receipt of hemoperfusion or systemic corticosteroid therapy within 3 months prior to enrollment; or (3) declined participation. Healthy controls were age-matched volunteers without significant acute or chronic illnesses. None of the healthy controls had received any vaccinations within 4 weeks prior to blood sampling.

### Data collection

Baseline clinical and demographic data were collected at enrollment. These included sex, age, and laboratory parameters: serum creatinine, blood urea nitrogen, complete blood count (white blood cell and neutrophil counts), immune profiles (C3, C4, IgA, IgG, IgM), and urinalysis markers (α1-microglobulin, immunoglobulin G, and 24-hour proteinuria). Renal histology reports were reviewed when available. Peripheral blood samples were then drawn to analyze γδ T cells, assessing their frequency, surface markers (including CD69 and NKG2D), and intracellular cytokines.

### Collecting and processing sample

Peripheral blood samples (3 mL) were collected from patients within 24 h of admission and from healthy controls under aseptic conditions. All samples were promptly transported to the laboratory for processing. Peripheral blood mononuclear cells (PBMCs) were isolated using standard Ficoll-Hypaque density gradient centrifugation.

### Flow cytometry

For surface marker analysis, PBMCs were stained with fluorochrome-conjugated antibodies against human CD3, TCR-γδ, CD69, NKG2D, TLR4, TRAIL, Fas-L, perforin, and granzyme B (all used at 1.25 µL/test; sourced from BioLegend, San Diego, CA, or BD Bioscience, Franklin Lakes, NJ, as applicable). For intracellular cytokine detection, cells were fixed, permeabilized, and stained with antibodies targeting IL-10, IL-17 A, and TNF-α (1.25 µL/test; BioLegend). Appropriate isotype controls were included in parallel. Samples were acquired on a flow cytometer (BD Biosciences) and analyzed using FlowJo software (Tree Star, Ashland, OR). The frequency, surface phenotype, and cytokine profile of γδ T cells (defined as CD3⁺TCR-γδ⁺ within PBMCs) were analyzed. The gating strategy is illustrated in Supplementary Figure S1. The reference range for leukocyte count (WBC: 4.3–11.3 × 10⁹/L) was derived from the local clinical laboratory standards.

### Statistical analysis

Statistical analyses were performed using SPSS 26 (IBM Corp, Armonk, NY). Continuous data are presented as mean±standard deviation or as median (interquartile range); categorical data are expressed as percentages. Group comparisons were conducted using the Student’s t-test, one-way ANOVA, chi-square test, Mann-Whitney U test, or Kruskal Wallis test with Dunn Bonferroni post hoc correction, as appropriate. A multivariate logistic regression model was used to identify variables associated with IgAVN. The predictive value of the IL-17 A⁺ γδ T cell percentage for progression from IgAV without nephritis (IgAVwoN) to IgAVN was evaluated using receiver operating characteristic curve analysis. In patients with IgAVN, the association between IL-17 A⁺ γδ T cell percentage and 24 h proteinuria was assessed via Pearson’s correlation coefficient. *P* < 0.05 was considered statistically significant (^*^P<0.05, ^**^P<0.01, ^***^P<0.001).

## Result

### Clinical characteristics of study subjects

A total of 88 children were enrolled and categorized into three groups: IgAVN (*n* = 39), IgAVwoN (*n* = 36), and healthy controls (HC, *n* = 13). The overall mean age was 8.84 ± 3.11 years, with 53.41% of participants being male. No significant intergroup differences were observed in age or sex. Children in the IgAVN group demonstrated significantly higher values than those in the IgAVwoN group across several renal parameters: serum creatinine (42.62 ± 16.47 vs. 34.53 ± 8.94 µmol/L, *P* = 0.011), urea nitrogen (4.65 ± 1.42 vs. 4.01 ± 1.14 mmol/L, *P* = 0.035), urinary IgA1 [0.78 (0.55–1.41) vs. 0.48 (0.20–0.95) mg/g Cr, *P* = 0.001], urinary microalbumin [1.52 (1.08–22.3) vs. 0.99 (0.62–1.27) mg/g Cr, *P* = 0.002], and 24-hour proteinuria [0.49 (0.02–0.53) vs. 0.02 (0.01–0.04) g/day, *P* = 0.001]. Other baseline characteristics are summarized in Table 1.

**Table 1:** Demographic and clinical characteristics of the study population on admission.

Variable	Total(*n* = 88)	IgAVwoN(*n* = 36)	IgAVN(*n* = 39)	HC(*n* = 13)	*P*
Age, yrs	8.84 ± 3.11	8.41 ± 2.86	9.04 ± 3.64	9.46 ± 1.77	0.509
Male, n (%)	47(53.41)	19(52.78)	22(56.41)	6(46.15)	
WBC, ×10^9^ /L	10.44 ± 4.45	10.45 ± 4.53	10.44 ± 4.43	–	0.989
Neutrophils, %	62.41 ± 14.44	60.74 ± 14.29	63.95 ± 14.59	–	0.340
IgG, g/L	10.11 ± 3.02	10.47 ± 3.21	9.78 ± 2.83	–	0.320
IgA, g/L	2.54 ± 1.13	2.68 ± 1.34	2.42 ± 0.88	–	0.316
IgM, g/L	1.15 ± 0.43	1.15 ± 0.41	1.16 ± 0.45	–	0.880
IgE, IU/ml	79.90(40.90,265)	65.85(14.18,249)	95.1(53.60,265)	–	0.056
C3, g/L	1.13 ± 0.22	1.12 ± 0.20	1.14 ± 0.24	–	0.676
C4, g/L	0.22 ± 0.08	0.22 ± 0.08	0.23 ± 0.79	–	0.565
ALB, g/L	41.62 ± 4.76	42.65 ± 3.46	40.67 ± 5.89	–	0.072
Renal involvement					
Serum creatinine, umol/L	38.73 ± 13.92	34.53 ± 8.94	42.62 ± 16.47	–	0.011
Urea nitrogen, mmol/L	38.73 ± 13.92	4.01 ± 1.14	4.65 ± 1.42	–	0.035
Urine examination (mg/g Cr)					
Microglobulin A1	0.59(0.42,1.19)	0.48(0.20,0.95)	0.78(0.55,1.41)	–	0.001
Immunoglobulin G	0.56(0.40,1.01)	0.51(0.40,0.77)	0.37(0.61,2.18)	–	0.191
Transferrin	0.20(0.20,0.50)	0.20(0.20,0.38)	0.20(0.20,0.85)	–	0.266
Microalbumin	1.10(0.70,1.78)	0.99(0.62,1.27)	1.52(1.08,22.3)	–	0.002
Proteinuria, g/day	0.04(0.02,0.07)	0.02(0.01,0.04)	0.49(0.02,0.53)	–	0.001

### Percentage and phenotypic changes of γδ T cells in the IgAV pediatric patients

**Fig. 1:**
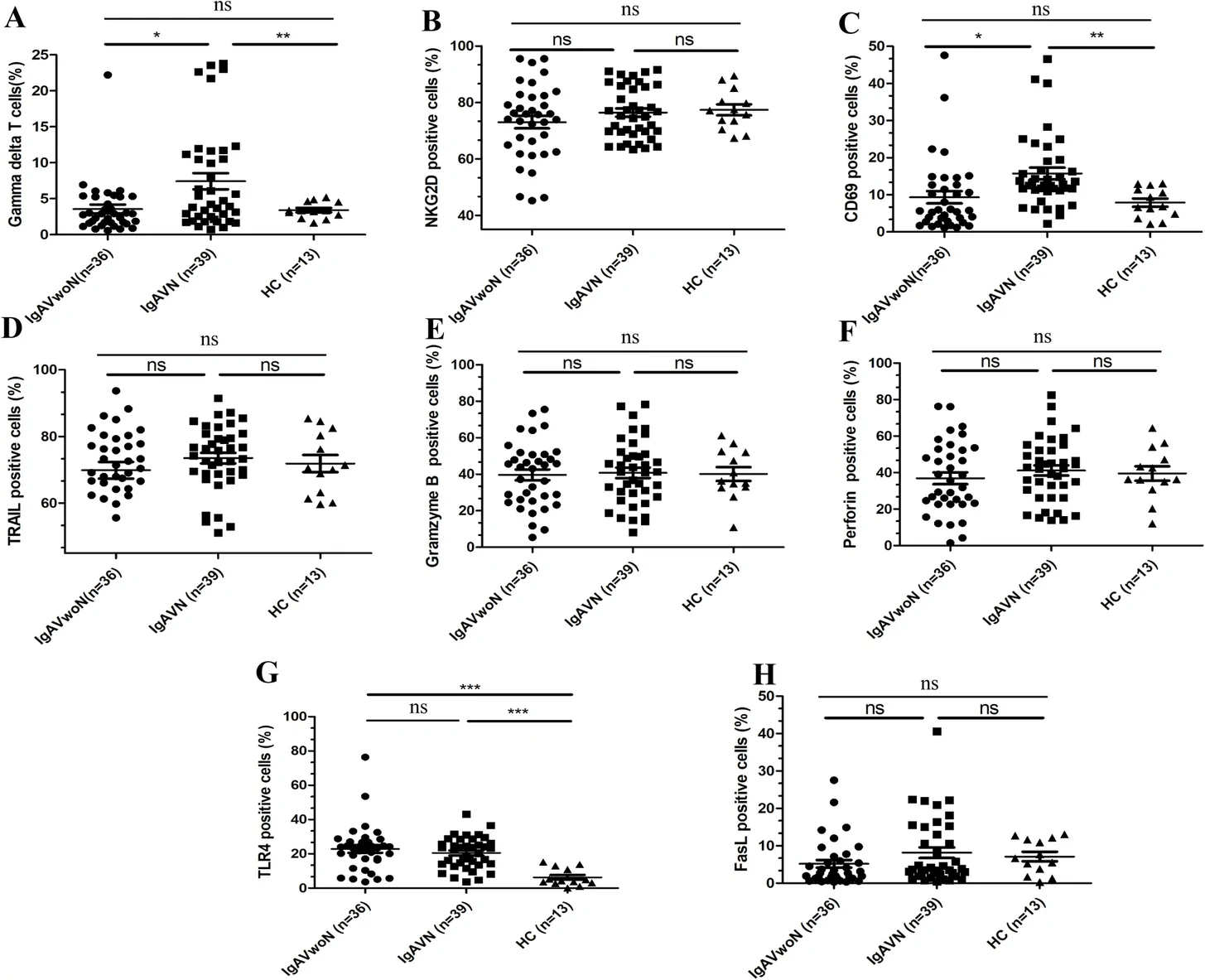
Percentage of γδ T cells and surface markers on γδ T cells in IgAVwoN, IgAVN and HC groups. Antibody staining was performed for the freshly prepared PBMCs. CD3^+^TCR-γδ T cells were gated and the percentage of positive cells was analyzed by and flow cytometry analysis. (**A**) percentage of γδ T cells in CD3^+^ cells; (**B**) percentage of NKG2D^+^ γδ T cells; (**C**) percentage of CD69^+^ γδ T cells; (**D**) percentage of TRAIL^+^ γδ T cells; (**E**) percentage of Granzyme B^+^ γδ T cells; (**F**) percentage of Perforin^+^ γδ T cells; (**G**) Percentage of TLR4^+^ γδ T cells; (**H**) percentage of FasL^+^ γδ T cells. PBMCs, peripheral blood mononuclear cell; γδ T: gamma delta T; CD, cluster of differentiation; NKG2D, natural-killer group 2 member D; TRAIL: Tumor necrosis factor related apoptosis inducing ligand; TLR4: Toll-like receptor 4; FasL: Factor related apoptosis ligand. ns, non-significant.

The proportion of γδ T cells within the CD3⁺ lymphocyte compartment was significantly higher in IgAVN patients compared to both IgAVwoN and HC groups (*P* < 0.05). To assess their functional state, we analyzed key surface markers: CD69 for early activation; NKG2D for co-stimulation; perforin and granzyme B for cytotoxicity; and TLR4, TRAIL, and FasL for apoptotic function. Compared to HCs, γδ T cells from IgAVN patients exhibited a significantly greater frequency of CD69⁺ and TLR4⁺ subsets (*P* < 0.01; Fig. 1C, G). Furthermore, the percentage of CD69⁺ γδ T cells was also elevated in the IgAVN group relative to the IgAVwoN group (*P* < 0.05). In contrast, no significant difference in TLR4 expression was observed between the two patient groups. As shown in Fig. 1, the expression levels of NKG2D, TRAIL, perforin, granzyme B, and FasL on γδ T cells did not differ significantly among the three cohorts (*P* > 0.05).

### Cytokine expression of γδ T cells

**Fig. 2:**
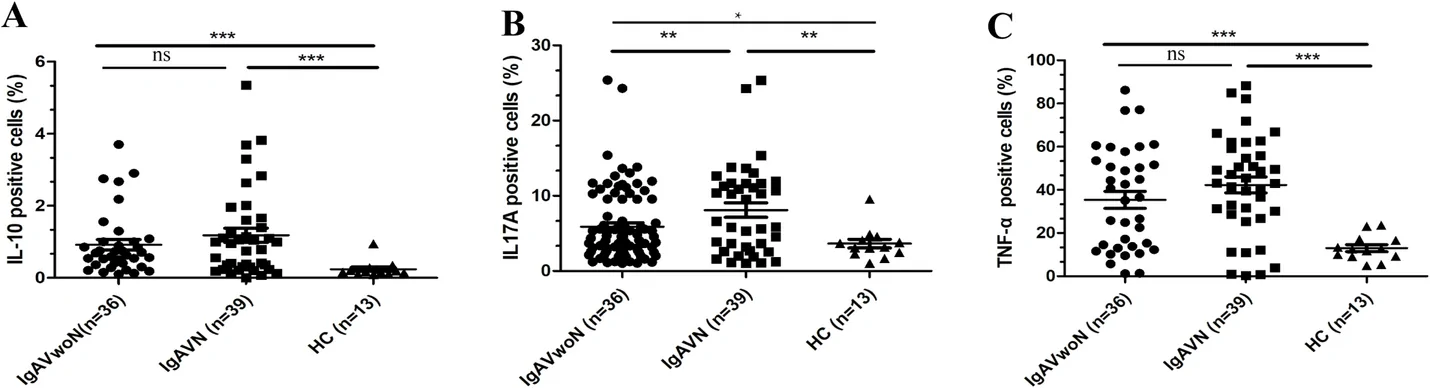
Cytokines expression on γδ T cells in IgAVwoN, IgAVN and HC groups. γδ T cells isolated from fresh blood were fixed, permeabilized, and stained forintracellular cytokines. CD3^+^ TCR-γδ cells were gated and cytokine expressions were analyzed using flow cytometry. (**A**) IL-10^+^ γδ T cells; (**B**) IL-17 A^+^ γδ T cells; (**C**) TNF-α^+^ γδ T cells. γδ T: gamma delta T; CD: cluster of differentiation; IL: interleukin; TNF-α: Tumor necrosis factor-α.

To further evaluate the pro-inflammatory and immunoregulatory functions of γδ T cells, we quantified their secretion of IL-17 A, IL-10, and TNF-α. Cytokine analysis revealed elevated levels of IL-10 and TNF-α in IgAVwoN patients relative to HC group (*P* < 0.05). IgAVN patients exhibited a marked increase in both pro-inflammatory cytokine (IL-17 A) and cytokines involved in immune regulation (IL-10 and TNF-α) compared to HC groups (*P* < 0.01). Additionally, IL-17 A levels were significantly higher in the IgAVN group than in the IgAVwoN group (Fig. 2**).**

### Independent risk factors for IgAVN

To identify factors independently associated with IgAVN, we performed multivariable logistic regression. Variables with a *P* value < 0.1 from univariate analyses, along with TLR4 expression and the cytokines IL-10, IL-17 A, and TNF-α, were included in the model. The analysis identified the percentage of IL-17 A⁺ γδ T cells as an independent risk factor for IgAVN (OR = 1.202, 95% CI 1.014–1.424; *P* = 0.034), as detailed in Table 2.

**Table 2:** Multi-logistic regression for risk factors of IgAVN in children.

Variable	B	SE	Wald	*P*	OR	95%CI	
						Lower	Upper
IgE	0.000	0.001	0.134	0.715	1.000	0.999	1.001
Serum creatinine	0.050	0.030	2.889	0.089	1.051	0.992	1.114
Urea nitrogen	0.347	0.254	1.876	0.171	1.415	0.861	2.326
Urinary microglobulin A1	0.039	0.143	0.076	0.783	1.040	0.786	1.376
Urinary microalbumin	0.126	0.119	1.136	0.286	1.135	0.899	1.432
TNF-α ^+^γδ T cells%	0.022	0.015	2.337	0.126	1.002	0.994	1.052
IL−10^+^γδ T cells%	0.201	0.316	0.407	0.524	1.223	0.659	2.270
IL−17 A^+^ γδ T cells%	0.184	0.087	4.513	0.034	1.202	1.014	1.424
TLR4^+^ γδ T cells%	−0.001	0.027	0.003	0.959	0.999	0.947	1.053

### The ability of IL-17 A^+^ γδ T to discriminate patients with IgAVN

**Fig. 3:**
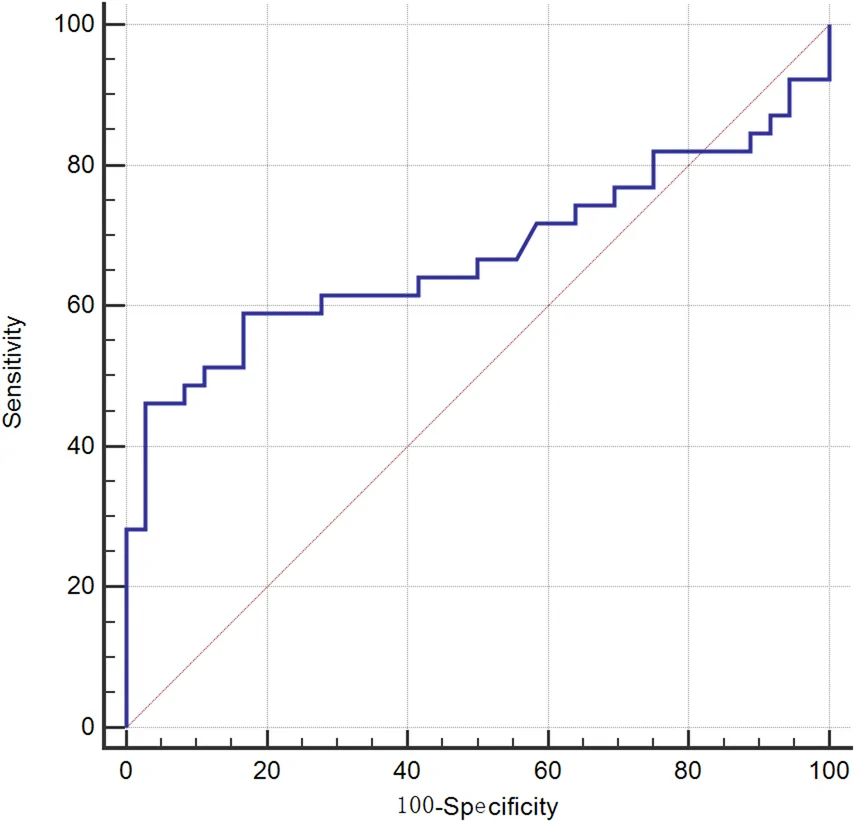
Receiver operating characteristic analysis of IL-17 A^+^ γδ T cells percentage for the prediction of IgAVN.

To assess its clinical predictive potential, the IL-17 A⁺ γδ T cell percentage was tested via ROC curve analysis for discriminating IgAVN. The results confirmed its role as a predictor, showing moderate accuracy (AUC 0.673, 95% CI 0.555–0.777) with a notably high specificity of 97.22% and a sensitivity of 46.15% (Fig. 3).

### Association between the number of IL-17 A^+^ γδ T Cells and 24-hour proteinuria in patients with IgAVN

**Fig. 4:**
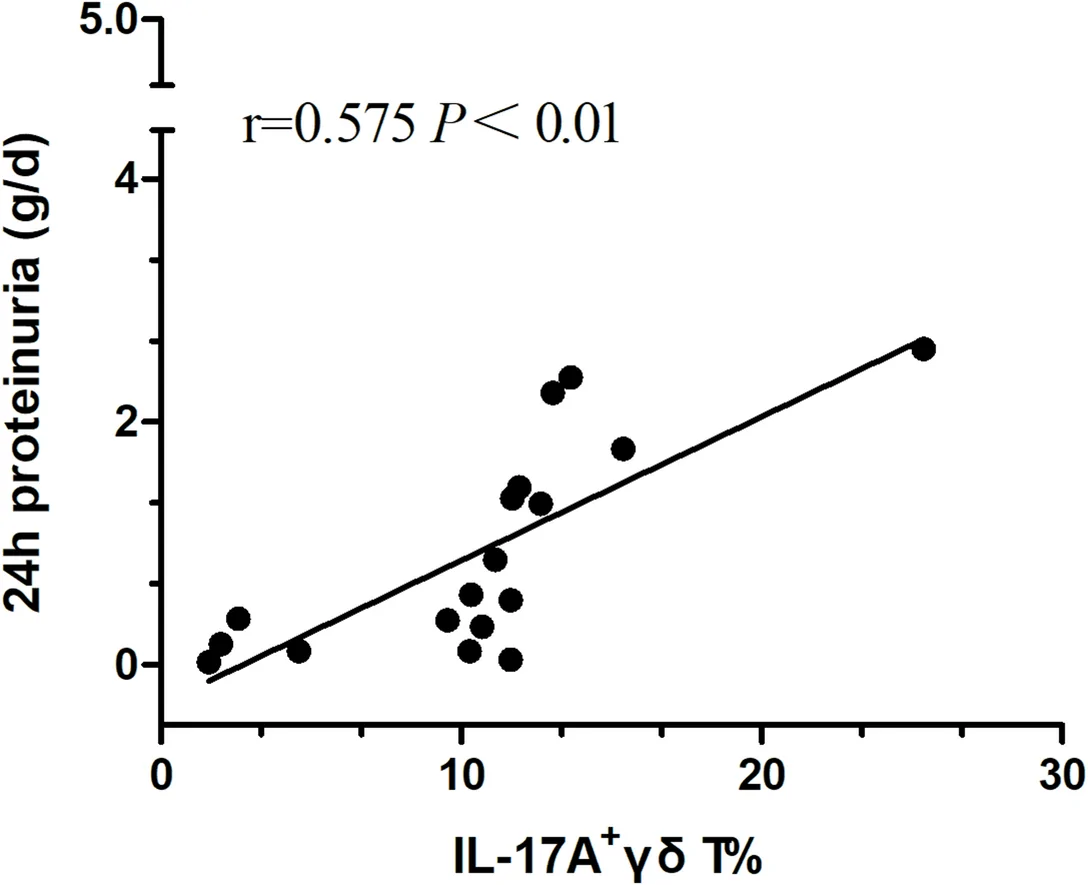
Positive correlation between the percentage of IL-17 A^+^γδ T cells and 24-hour proteinuria in the IgAVN cohort.

Given that proteinuria is an established independent risk factor for renal impairment, and our finding that IL-17 A^+^γδ T cells are an independent risk factor for IgAVN, we explored the association between this cellular subset and 24-hour proteinuria in IgAVN patients. Patients were categorized into those with (*n* = 18) and without (*n* = 21) significant proteinuria. Compared to the non-proteinuric group, proteinuric patients had significantly lower serum albumin (42.65 ± 3.46 vs. 38.43 ± 6.75 g/L, *P* = 0.019), a higher percentage of IL-17 A^+^ γδ T cells (6.01 ± 5.59% vs. 10.54 ± 5.55%, *P* = 0.016), and higher 24-hour proteinuria [0.55 (0.16–1.54) vs. 0.03 (0.01–0.04) g/day, *P* = 0.001] (Table 3). Age and gender did not differ between groups. Furthermore, correlation analysis revealed a statistically significant positive relationship between the percentage of IL-17 A^+^γδ T cells and the level of 24-hour proteinuria (*r* = 0.575, *P* < 0.01) (Fig. 4).

**Table 3:** Patient characteristics at baseline in IgAVN patients with or without proteinuria.

Variable	IgAVN (*n* = 39)	Without protienuria (*n* = 21)	With protienuria (*n* = 18)	*P*
Age, yrs	9.04 ± 3.64	8.03 ± 3.50	10.22 ± 3.54	0.060
Male, n (%)	22(56.41)	12(57.14)	10(55.56)	0.921
WBC, ×10^9^ /L	10.44 ± 4.43	10.32 ± 3.52	10.56 ± 5.41	0.873
Neutrophils, %	63.95 ± 14.59	64.57 ± 13.65	63.21 ± 15.97	0.775
IgG, g/L	9.78 ± 2.83	10.45 ± 2.07	8.99 ± 3.42	0.110
IgA, g/L	2.42 ± 0.88	2.38 ± 0.86	2.47 ± 0.88	0.729
IgM, g/L	1.16 ± 0.45	1.15 ± 0.41	1.16 ± 0.93	0.982
IgE, IU/ml	95.10(53.60,265.00)	186.00(60.75,350.50)	88.20(50.93,172.00)	0.056
C3, g/L	1.14 ± 0.24	1.14 ± 0.24	1.14 ± 0.24	0.942
C4, g/L	0.23 ± 0.79	0.23 ± 0.07	0.23 ± 0.90	0.934
ALB, g/L	40.67 ± 5.89	38.43 ± 6.75	42.65 ± 3.46	0.019
IL−17 A^+^ γδ T cells%	8.01 ± 5.96	6.01 ± 5.59	10.54 ± 5.55	0.016
Renal involvement				
Serum creatinine, umol/L	42.62 ± 16.47	40.57 ± 13.39	45.00 ± 19.60	0.410
Urea nitrogen, mmol/L	4.65 ± 1.42	4.56 ± 1.54	5.97 ± 4.73	0.205
Proteinuria, g/day	0.49(0.02,0.53)	0.03(0.01,0.04)	0.55(0.16,1.54)	0.001

## Discussion

The study establishes an association between circulating IL-17 A^+^γδ T cells and pediatric IgAVN. Our data demonstrated that this specific cell subset was expanded, displayed an early activation phenotype, and served as an independent risk factor for IgAVN. Despite some data dispersion and potential outliers observed in Fig. 4, the percentage of this subset exhibited a significant positive correlation with 24-hour proteinuria (*r* = 0.575), a key clinical marker of glomerular injury. Our results were consistent with a previous study conducted by Guo e tal^18^.

The enhanced CD69 expression observed on γδ T cells from IgAVN patients points to their active participation in the disease process. Critically, we identified the IL-17 A producing subset within these cells as an independent risk factor for nephritis (AUC, 0.673; 95% CI, 0.555–0.777; *P*<0.05). Although an AUC of 0.673 suggests limited diagnostic value when this marker is used alone, and the wide confidence interval reflects our modest sample size, this finding is consistent with the recognized pathogenic role of IL-17 A in glomerular diseases^5–8^, indicating that it still possesses certain clinical value. This aligns with the recognized pathogenic role of IL-17 A in other glomerular diseases, where it facilitates neutrophil recruitment and podocyte damages^5–8^, establishing γδ T cells as a relevant source of this cytokine in IgAVN.

Although γδ T cells possess cytotoxic potential via mediators like Fas/FasL, TRAIL, perforin/granzyme, and NKG2D^19^, our data showed no significant change in the expression of these markers in IgAVN, arguing against a major role for direct cytotoxicity in renal injury. A more relevant finding was the elevated TLR4 on γδ T cells in IgAV patients versus controls, suggestive of activation by damage-associated molecular patterns. However, this TLR4 upregulation was similar in patients without nephritis (IgAVwoN), linking it to systemic immune dysregulation in IgAV rather than to kidney specific pathogenesis. Importantly, a distinct increase in IL-17 A production was observed specifically in γδ T cells from IgAVN patients. This pattern aligns with established inflammatory axes (e.g., HMGB1-TLR4-IL-23-IL-17 A) shown to mediate tissue injury in other organ systems^20–23^. Furthermore, analogous γδ T cell-dependent, IL-17-driven pathways are implicated in fostering pathological inflammation in contexts such as transplant rejection and tumor radioresistance^9,24,25^. Collectively, our findings suggest that in IgAVN, γδ T cells likely contribute to renal inflammation not through canonical cytotoxicity, but via the mechanism involving TLR4 sensing and subsequent IL-17 A release.

Proteinuria is a recognized independent risk factor for renal impairment^26^, and has been shown to promote disease progression in IgA nephropathy^27,28^. In our cohort, the percentage of IL-17 A^+^ γδ T cells correlated positively with 24-hour proteinuria levels (*r* = 0.575). While approximately half of the IgAVN patients in our study presented with hematuria without significant proteinuria, the association with this immune subset suggests it retains predictive value for identifying nephritis.

Several limitations must be acknowledged. The cross-sectional, single-center design and modest sample size limit generalizability and preclude causal inference. The limited sample size also constrains the stability of our ROC and correlation analyses. Additionally, the absence of longitudinal data hinders assessment of how this cellular signature evolves from the acute phase to remission or with therapy. Furthermore, whether the elevation of circulating IL-17 A⁺ γδ T cells is unique to IgAV or also occurs in other childhood vasculitides such as Kawasaki disease remains unknown, although both diseases share genetic and immunological features while differing in vessel involvement and organ tropism^29^. Moreover, our study did not include patients with IgA nephropathy. Future comparative studies including multiple vasculitis entities as well as IgA nephropathy are needed to determine whether this cellular signature is specific to IgAVN or represents a shared feature of IgA-associated glomerulonephritides. Despite these constraints, our findings delineate a novel immune correlate of IgAVN and provide a foundation for future investigative and translational work.

## Conclusion

This study identified circulating IL-17 A⁺ γδ T cells as an expanded, activated subset that independently predicts renal involvement in pediatric patients with IgAV. The percentage of IL-17 A⁺ γδ T cells correlates with proteinuria severity. While Th17 cells remain the dominant source of IL-17, IL-17 A⁺ γδ T cells may represent one of several potential sources of this cytokine. Further investigation is needed to determine their precise contribution to IgAVN pathogenesis and the potential of the IL-17 A pathway as a therapeutic target.

## Supplementary Information

Below is the link to the electronic supplementary material.


Supplementary Material 1


## Data Availability

All data used during the current study are available from corresponding author upon reasonable request.
