# Supplementary material for: Activation of circulating γδ T cells in pediatric IgA vasculitis nephritis links the IL-17 A+ subset to renal risk
Source: Sci Rep. 2026 Jun 15;16:27286. doi: 10.1038/s41598-026-57972-7 (PMC13530118; doi:10.1038/s41598-026-57972-7)
Supplement: Supplementary file 1 [file 41598_2026_57972_MOESM1_ESM.docx]

**Supplementary Fig. 1. A flow chart for processing and examining sample**

**Supplementary Fig. 2. Flow cytometry plot and gate for** **percentage of γδ T cells**

**Supplementary Fig. 3.** **An example for the gating strategy for surface and intracellular markers production by γδ T cells exported from Flowjo software**
